# Comprehensive analysis of abnormal expression, prognostic value and oncogenic role of the hub gene FN1 in pancreatic ductal adenocarcinoma *via* bioinformatic analysis and *in vitro* experiments

**DOI:** 10.7717/peerj.12141

**Published:** 2021-09-06

**Authors:** Xiaohua Lei, Guodong Chen, Jiangtao Li, Wu Wen, Jian Gong, Jie Fu

**Affiliations:** 1 The First Affiliated Hospital, Department of Hepato-Biliary-Pancreatic Surgery, Hengyang Medical School, University of South China, Hengyang, Hunan, China; 2 Department of Gastroenterology, The Second Xiangya Hospital, Central South University, Changsha, Hunan, China; 3 Department of General Surgery, The Second Xiangya Hospital, Central South University, Changsha, Hunan, China

**Keywords:** Pancreatic ductal adenocarcinoma, FN1, Bioinformatic analysis, *I. vitro* experiments

## Abstract

**Background:**

Pancreatic ductal adenocarcinoma (PDAC) is one of the most commonly diagnosed cancers with a poor prognosis worldwide. Although the treatment of PDAC has made great progress in recent years, the therapeutic effects are still unsatisfactory. Methods. In this study, we identified differentially expressed genes (DEGs) between PDAC and normal pancreatic tissues based on four Gene Expression Omnibus (GEO) datasets (GSE15471, GSE16515, GSE28735 and GSE71729). A protein–protein interaction (PPI) network was established to evaluate the relationship between the DEGs and to screen hub genes. The expression levels of the hub genes were further validated through the Gene Expression Profiling Interactive Analysis (GEPIA), ONCOMINE and Human Protein Atlas (HPA) databases, as well as the validation GEO dataset GSE62452. Additionally, the prognostic values of the hub genes were evaluated by Kaplan–Meier plotter and the validation GEO dataset GSE62452. Finally, the mechanistic roles of the most remarkable hub genes in PDAC were examined through *in vitro* experiments.

**Results:**

We identified the following nine hub genes by performing an integrated bioinformatics analysis: COL1A1, COL1A2, FN1, ITGA2, KRT19, LCN2, MMP9, MUC1 and VCAN. All of the hub genes were significantly upregulated in PDAC tissues compared with normal pancreatic tissues. Two hub genes (FN1 and ITGA2) were associated with poor overall survival (OS) rates in PDAC patients. Finally, *in vitro* experiments indicated that FN1 plays vital roles in PDAC cell proliferation, colony formation, apoptosis and the cell cycle.

**Conclusions:**

In summary, we identified two hub genes that are associated with the expression and prognosis of PDAC. The oncogenic role of FN1 in PDAC was first illustrated by performing an integrated bioinformatic analysis and *in vitro* experiments. Our results provide a fundamental contribution for further research aimed finding novel therapeutic targets for overcoming PDAC.

## Introduction

Pancreatic ductal adenocarcinoma (PDAC) is one of the cancers with worst prognosis, with a 5-year survival rate of less than 5% and a median survival time of less than 6 months after diagnosis (Jemal et al., 2011; Zhang et al., 2019; Zhou et al., 2017). Because of the non-specificity of symptoms, most PDAC patients are diagnosed at an advanced stage. Even patients whose disease is detected early, and who receive radical surgery followed by chemotherapy, have a median survival rate of 2 years and a 5-year survival rate of 15–20% (Zhou et al., 2017).

Although we are constantly updating and deepening our understanding of the complex genomic, epigenetic, and metabolomic alterations, and complex interplay between tumor cells and immune cells, mesenchymal cells, and epithelial cells in PDAC, unfortunately, patient quality of life and survival time have not improved significantly (Neoptolemos et al., 2018). It is currently believed that the development of PDAC is caused by multiple stages of gene mutations, including KRAS, TP53, SMAD4 and CDKN2A (Biankin et al., 2012; Kamisawa et al., 2016; Zhang et al., 2019). However, no effective therapeutic drugs have been developed for these targets. To explore effective biomarkers and therapeutic drugs for cancers, high-throughput sequencing and chips are widely used worldwide (Kristensen et al., 2019; Lusito et al., 2019; Massard et al., 2017). At the same time, combined with bioinformatics analysis and experimental study, valuable biological information was discovered that was useful for cancer treatment (Jiang et al., 2019; Li et al., 2020; Li et al., 2018; Xu et al., 2020). In recent years, some genes have been reported to play oncogenic roles in pancreatic cancer, but the clinical sample size enrolled in these studies is not enough, and the biological role of these genes in PDAC has not been fully studied (Okada et al., 2021; Wang et al., 2019; Zhou et al., 2019).

In this study, differentially expression genes (DEGs) between pancreatic ductal adenocarcinoma and normal pancreatic tissues were explored using four Gene Expression Omnibus (GEO) datasets, and protein–protein interaction (PPI) network was constructed through the Search Tool for the Retrieval of Interacting Genes (STRING) database based on these DEGs. Next, enrichment analysis was applied to these DEGs to explore the underlying mechanisms of PDAC. Furthermore, hub genes were screened by integrated analysis of the correlation between these DEGs, and their expression levels and prognostic values in PDAC were detected by online databases including Gene Expression Profiling Interactive Analysis (GEPIA), ONCOMINE, the Human Protein Atlas (HPA), the Kaplan–Meier Plotter and the validation GEO dataset. Finally, the biological roles of the most remarkable hub gene in PDAC, FN1, were further investigated through *in vitro* experiments.

## Materials & Methods

### Gene expression profile data

Microarray data of PDAC patients were obtained from the GEO database (http://www.ncbi.nlm.nih.gov/geo/). In total, 262 pancreatic ductal adenocarcinoma samples and 143 normal samples were enrolled in our study. The GSE15471 dataset includes 36 PDAC samples and matching noncancerous samples. GSE16515 consists of 36 PDAC samples and 16 noncancerous samples. GSE28375 includes 45 PDAC samples and matching noncancerous samples. GSE71729 consists of 145 PDAC samples and 46 noncancerous samples. GSE62452 contains 69 PDAC samples and 61 noncancerous samples. In this study, GSE15471, GSE16515, GSE28375 and GSE71729 were used as discovery datasets, and GSE62452, which contains survival data was used as a validation dataset. The gene expression profile data characteristics are listed in Table 1.

**Table 1:** The gene expression profile data characteristics.

Reference	PMID	Record	Tissue	Platform	Tumor	Normal
Badea et al.	19260470	GSE15471	PDAC	[HG-U133_Plus_2] Affymetrix Human Genome U133 Plus 2.0 Array	36	36
Pei et al.	19732725	GSE16515	PDAC	[HG133_Plus_2] Affymetrix Human Genome U133 Plus 2.0 Array	36	16
Zhang et al.	22363658	GSE28735	PDAC	[HuGene-1_0-st] Affymetrix Human Gene 1.0 ST Array [transcript (gene) version]	45	45
Moffitt et al.	26343385	GSE71729	PDAC	Agilent-014850 Whole Human Genome Microarray 4 × 44 K	145	46
Yang et al.	27197190	GSE62452	PDAC	[HuGene-1_0-st] Affymetrix Human Gene 1.0 ST Array [transcript (gene) version]	69	61

### DEGs extraction

The DEGs between PDAC and corresponding normal samples in each microarray were screened using the limma package (Ritchie et al., 2015) in R software. DEGs with a |log2FC| > 1 and adjusted *P*-value < 0.05 in each GEO dataset were considered statistically significant. Gene integration for the DEGs identified from the four discovery datasets was conducted by Robust Rank Aggregation (RRA) (Kolde et al., 2012) method.

### PPI network construction and hub gene identification

The STRING online database, which is a database of known and predicted protein interactions, was applied to construct the PPI network of DEGs, and only interactions with a combined score >0.4 were considered statistically significant (Szklarczyk et al., 2015). Moreover, Cytoscape software was used to visualize the PPI network (Shannon et al., 2003). Five methods in the plugin cytoHubba of Cytoscape software were used to evaluate the relationship between genes in the PPI network, including Betweenness, Closeness, Degree, EcCentricity and Radiality. The combination of these five methods is a common screening method of hub genes, which helps to improve the stability (Chin et al., 2014; Wu et al., 2019). The top 40 genes found by each method were intersected to find hub genes.

### Functional enrichment analysis

The ClusterProfiler package in R software was used for functional enrichment analysis. The functional enrichment analysis for the upregulated and downregulated DEGs, included molecular function (MF), biological process (BP), cellular component (CC) and Kyoto Encyclopedia of Genes and Genomes (KEGG) pathway analyses. A Q-value < 0.05 was considered statistically significant.

### Validation of the expression levels of the hub genes

To validate the mRNA expression levels of the screened hub genes between PDAC and normal pancreatic tissues, the online tool GEPIA (http://gepia.cancer-pku.cn/) (Tang et al., 2017), ONCOMINE database (https://www.oncomine.org/) (Rhodes et al., 2007) and validation dataset GSE62452 were used. The protein expression levels of the hub genes were determined though the HPA database (https://www.proteinatlas.org/) (Ouyang et al., 2019).

### Survival analysis of the hub genes

The effects of the hub genes on overall survival (OS) were determined by the web-based Kaplan–Meier (KM) plotter tool (https://kmplot.com/analysis/) and the survival data of the validation dataset GSE62452. PDAC patients were divided into two groups (“High expression” and “Low expression”) based on the best cutoff calculated automatically. The hazard ratios (HR) with 95% confidence intervals and log rank P-values were calculated and are shown on the plot (Buddhan et al., 2007).

### Cell culture and transfection

The human PDAC cell lines PANC1 and SW1990 were purchased from the Type Culture Collection of the Chinese Academy of Sciences (Shanghai, China). All cell lines were maintained in Dulbecco’s modified Eagle medium (HyClone, Logan, UT, USA) supplemented with 10% fetal bovine serum, 1% penicillin and streptomycin (Gibco, New York, NY, US). Cells were cultured at 37 °C in an atmosphere containing 5% CO2. Small interfering RNAs (siRNAs) targeting FN1 and a scrambled siRNA used as a normal control (NC) were purchased from RiboBio (Guangzhou, China). Transfection was conducted by Lipofectamine 2,000 Reagent (Invitrogen, Carlsbad, CA, USA) according to the manufacturer’s instructions. Briefly, PDAC cells were seeded into six-well plates (100,000 cells/well) in 1 mL of complete medium. After culturing for 12 h, siRNA targeting FN1 mixed with the same volume of Lipofectamine 2,000 was added to the wells. The final concentration of siFN1 is 100 nM. After 72 h, the transfected cells were harvested for further study.

### Colony formation assay

PDAC cells were seeded into six-well plates (1,000 cells/well) in 1.5 mL of complete medium. After culturing for 12 days, the colonies were stained with 0.5% crystal violet, and the number of colonies was counted.

### Cell counting kit-8 (CCK-8) assay

Cell proliferation viability was assessed by CCK-8 assay (Dojindo, Japan). Briefly, PDAC cells were seeded into 96-well plates at a density of 2,000 cells/well with 100 μl of culture medium. A total of 10 μl of CCK-8 reagent was added to each well at the indicated time points (0, 24, 48 and 72 h). The plates were incubated in the dark at 37 °C for 2 h, and then the optical density was measured at 450 nm. The experiments were performed in triplicate.

### 5-Ethynyl-2′-deoxyuridine (EdU) assay

EdU immunofluorescence staining was conducted using an EdU Kit provided by RiboBio (Guangzhou, China) according to the manufacturer’s instructions. In brief, suspended PDAC cells with different treatments were seeded into 96-well plates (2,000 cells/well) overnight. Then the cells were incubated with EdU for 2 h at 37 °C. After fixation in 4% paraformaldehyde for 10 min, the cells in plates were permeabilized with 0.5% Triton X-100. Next, the cells were stained with 1X Apollo reaction solution for 30 min. Finally, the nuclei were stained with Hoechst for 30 min and then imaged on a fluorescence microscope.

### Quantitative real-time PCR (qRT-PCR)

Total RNA was extracted from PDAC cells using RNAiso Plus Reagent (Takara, Japan). qRT-PCR and data analysis were performed as previously described (Fu et al., 2021). Specifically, GAPDH was used as an internal control. Relative expression levels of FN1 were calculated according to the 2^−ΔΔCt^ method. All of the primers in this study were synthesized by ShenGong (Shanghai, China). The FN1 primer sequences were as follows: (forward) 5′- TGGGCAACTCTGTCAACGAA-3′ and (reverse) 5′- GCCATTTTCTCCCTGACGG -3′. The GAPDH primer sequences were as follows: (forward) 5′-GCGACTTCAACAGCAACTCCC-3′ and (reverse) 5′-CACCCTGTTGCTGTAGCCGTA-3′.

### Flow cytometry

Cell cycle were measured by flow cytometry. Flow cytometry was performed as previously described (Fu et al., 2021). Briefly, PDAC cells after treatments were fixed in 70% ethanol at −20 °C overnight. The next day, the cells were stained with PI (Sigma–Aldrich, St. Louis, MO, USA) for 20 min at room temperature in the dark and analyzed by flow cytometry.

### Western blotting

Total cell lysates were extracted by radioimmunoprecipitation assay (RIPA) buffer containing 1 mM phenylmethanesulfonyl fluoride (PMSF). The protein concentration was detected by BCA method, and 25 μg of protein from cell lysates were loaded onto the gel in each group. Western blotting was performed as previously described(Zhu et al., 2020). The antibodies used in this study are listed: BAX (proteintech, 1:5,000), Bcl2 (proteintech, 1:1,000), caspase9 (proteintech, 1:500), cyclinD1 (proteintech, 1:1,000). Actin (proteintech, 1:5,000) was used as an internal reference.

### Gene set enrichment analysis (GSEA)

The ClusterProfiler package in R software was used for GSEA. Both of discovery datasets (GSE15471, GSE16515, GSE28375 and GSE71729) and validation dataset GSE62452 were included in GSEA (gene sets “KEGG_CELL_CYCLE” and “KEGG_APOPTOSIS”), which was performed between tumors and normal tissues.

### Statistical analysis

SPSS was used to analyze the data. All data were expressed as the mean ± SD. Student’s t-test was used in the two-group comparisons, and one-way ANOVA was used for more than two groups. Differences were considered significant at a *P* value < 0.05.

## Results

### Identification of DEGs in PDAC microarray datasets and construction of the PPI network

**Figure 1:**
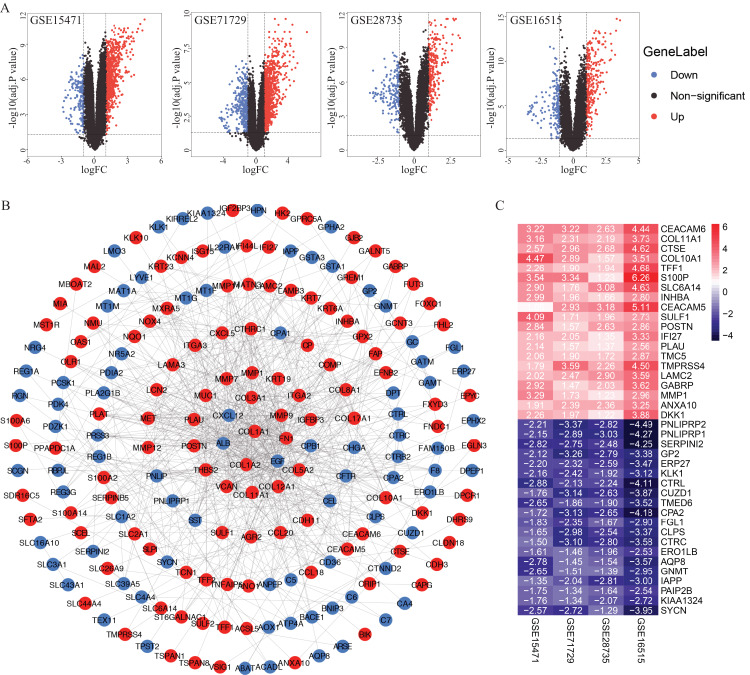
Identification of DEGs in PDAC microarray datasets and PPI network construction. (A) Volcano plot of the DEGs in GSE15471, GSE16515, GSE28735 and GSE71729 datasets. (B) PPI network of the 233 intersected DEGs. (C) Heat map of the top 20 upregulated and downregulated DEGs. Upregulated genes were marked in red, downregulated genes were marked in blue. DEGs, differentially expressed genes; PDAC, pancreatic ductal adenocarcinoma; PPI, protein‑protein interaction.

A total of 922, 1,359, 415 and 460 DEGs from the GSE15471, GSE16515, GSE28735 and GSE71729 datasets were identified after standardization of the microarray results (Fig. 1A). A total of 233 genes overlapped among the four datasets consisting of 102 downregulated genes and 131 upregulated genes identified by the RRA method. The PPI network of the 233 DEGs was constructed by the STRING database and visualized by Cytoscape (Fig. 1B). According to the cut-off criteria, the top 20 differentially expressed upregulated and downregulated genes are shown in Fig. 1C.

### Functional and pathway enrichment analyses

**Figure 2:**
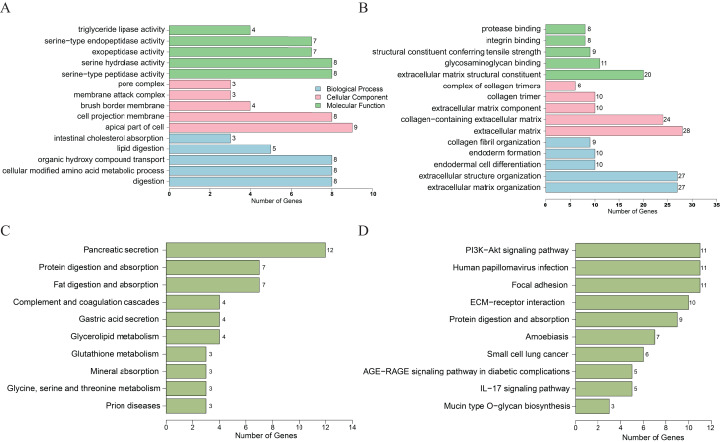
DEGs analyzed by GO enrichment (A, B) and KEGG enrichment (C, D) according to downregulation (A, C) or upregulation (B, D) of genes. DEGs, differentially expressed genes; GO, gene ontology. Y axis represents GO or KEGG pathway items; X axis represents the number of genes.

For the downregulated DEGs, GO analysis results (Fig. 2A) revealed that the biological processes (BPs) of DEGs were significantly changed in intestinal cholesterol absorption, lipid digestion, organic hydroxy compound transport, cellular modified amino acid metabolic process, etc. The DEGs were mainly enriched in cellular components (CCs) including pore complexes, membrane attack complexes, brush border membranes, cell projection membranes, etc. The DEGs were mainly enriched in molecular functions (MFs) including triglyceride lipase activity, serine-type endopeptidase activity, exopeptidase activity, serine hydrolase activity, etc. Enriched KEGG pathways (Fig. 2C) included pancreatic secretion, protein digestion and absorption, fat digestion and absorption, complement and coagulation cascades, glycerolipid metabolism, etc. For the upregulated genes, GO analysis results revealed (Fig. 2B) that the biological processes (BPs) of DEGs were significantly changed in collagen fibril organization, endoderm formation, endodermal cell differentiation, extracellular structure organization, etc. The DEGs were mainly enriched in cellular components (CCs) including complexes of collagen trimers, collagen trimers, extracellular matrix components and, etc. The DEGs were mainly enriched in molecular functions (MFs) including protease binding, integrin binding, extracellular matrix structural constituent conferring tensile strength, glycosaminoglycan binding, etc. KEGG pathways (Fig. 2D) included the PI3K-AKT signaling pathway, human papillomavirus infection, focal adhesion, ECM-receptor interaction, protein digestion and absorption, etc.

### Identification of the hub genes

We then evaluated the relationship between DEGs in the PPI network to select hub genes using five methods including Betweenness, Closeness, Degree, EcCentricity and Radiality. The top 40 genes found by each method were intersected to identify the hub genes. Thereafter, five downregulated genes (ALB, EGF, CFTR, CXCL12 and SST) and nine upregulated genes (MUC1, MMP9, KRT19, COL1A1, COL1A2, LCN2, ITGA2 and VCAN) in PDAC tissues were identified.

### Validation of the expression levels of the hub genes

**Figure 3:**
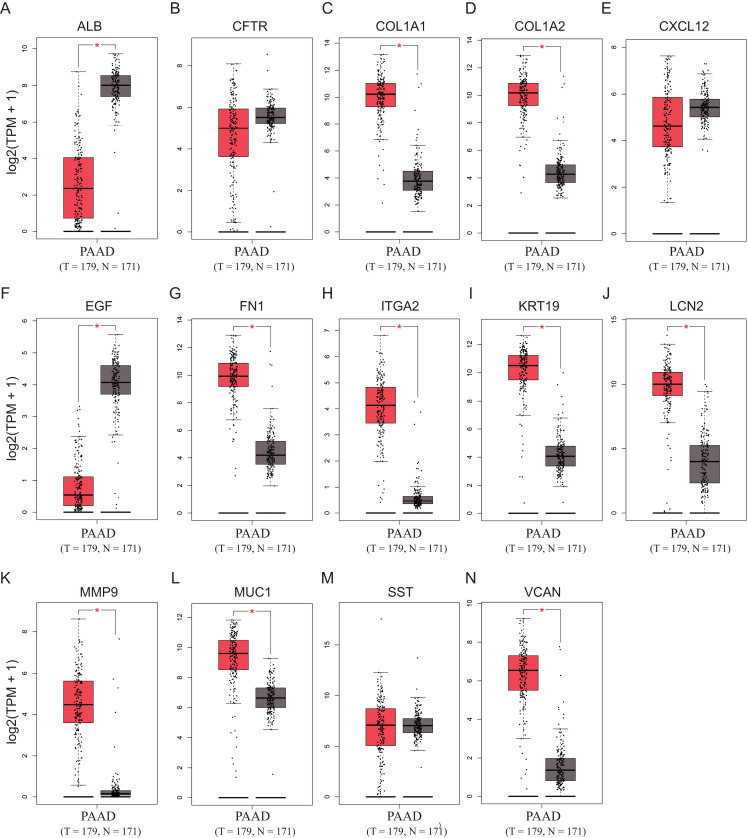
Validation of the mRNA expression levels of (A) ALB, (B) CFTR, (C) COL1A1, (D) COL1A2, (E) CXCL12, (F) EGF, (G) FN1, (H) ITGA2, (I) KRT19, (J) LCN2, (K) MMP9, (L) MUC1, (M) SST and (N) VCAN in pancreatic cancer tissues compared with normal pancreatic tissue. *P < 0.05 was considered statistically significant. The expression level is described by log2(TPM+1). Red represents tumor (*n* = 179), brown represents nontumor (*n* = 171).

**Figure 4:**
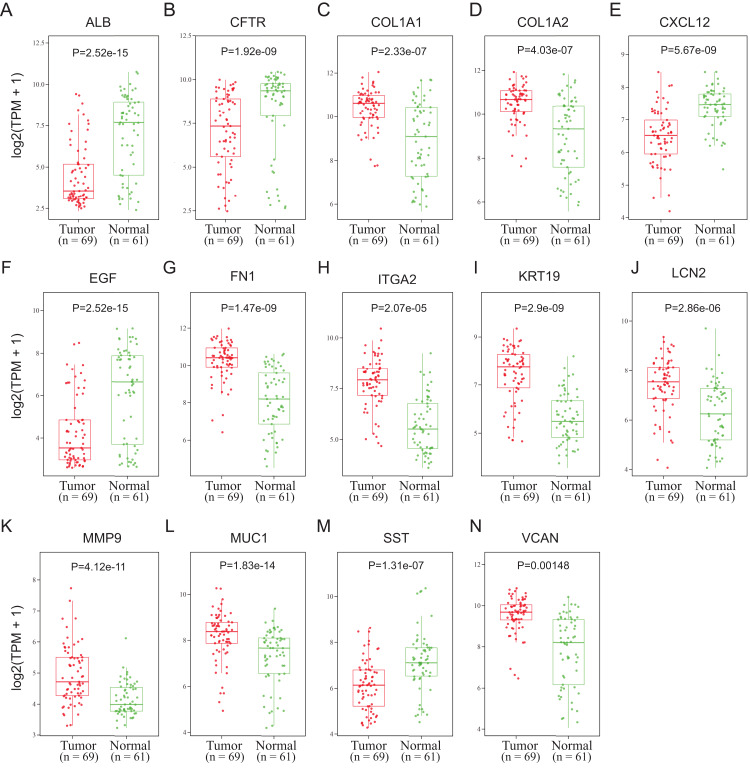
Validation of the mRNA expression levels of (A) ALB, (B) CFTR, (C) COL1A1, (D) COL1A2, (E) CXCL12, (F) EGF, (G) FN1, (H) ITGA2, (I) KRT19, (J) LCN2, (K) MMP9, (L) MUC1, (M) SST and (N) VCAN in pancreatic cancer tissues compared with normal pancreatic tissue. The expression level is described by log2(TPM+1). Red represents tumor (*n* = 69), green represents nontumor (*n* = 61).

**Figure 5:**
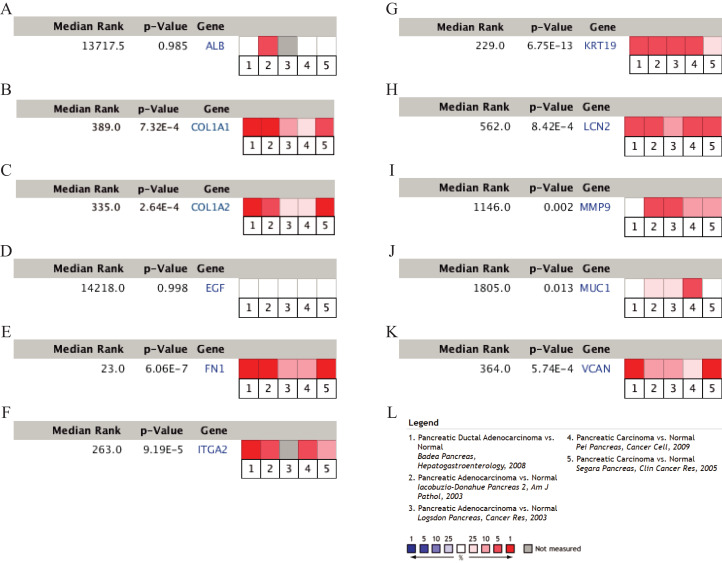
A meta-analysis of the mRNA expression levels of (A) ALB, (B) COL1A1, (C) COL1A2, (D) EGF, (E) FN1, (F) ITGA2, (G) KRT19, (H) LCN2, (I) MMP9, (J) MUC1 and (K) VCAN in PDAC tissues compared with normal pancreatic tissues by ONCOMINE. The colored squares represent the median rank of these genes across five datasets in ONCOMINE. *P*-value < 0.05 was considered as statistically significant. PDAC, pancreatic ductal adenocarcinoma.

**Figure 6:**
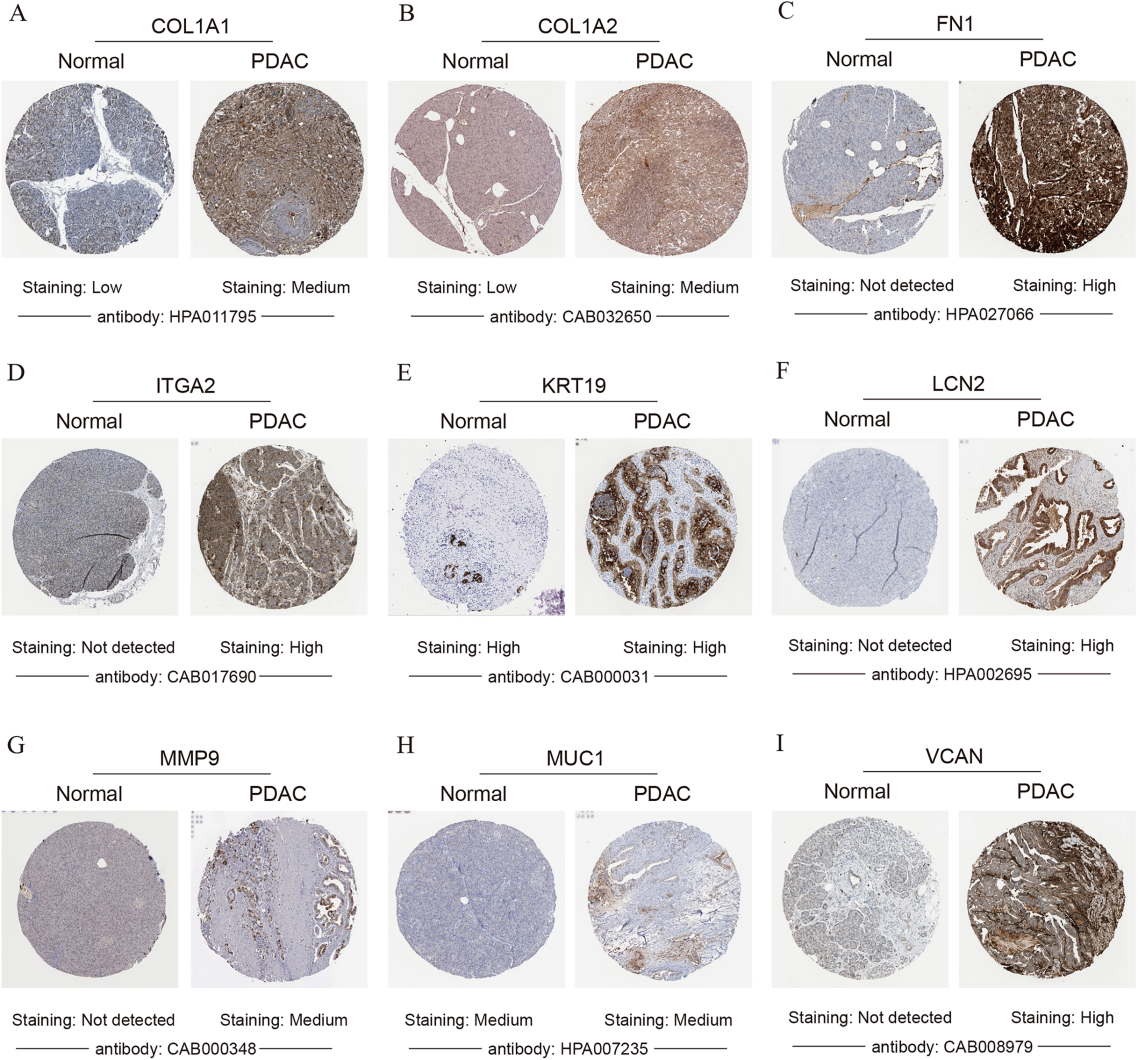
Validation of the protein expression levels of (A) COL1A1, (B) COL1A2, (C) FN1, (D) ITGA2, (E) KRT19, (F) LCN2, (G) MMP9, (H) MUC1 and (I) VCAN in PDAC tissues compared with normal pancreatic tissues by HPA database. PDAC, pancreatic ductal adenocarcinoma; HPA, human protein atlas.

To validate the mRNA expression levels of the 14 screened hub genes between PDAC and normal pancreatic tissues, the online tool GEPIA based on the TCGA (https://portal.gdc.cancer.gov/) (Sidaway, 2017) was used firstly. The results showed that 11 hub genes were significantly differentially expressed in PDAC tissues compared with normal pancreatic tissues (Fig. 3). Next, the validation dataset GSE62452 further confirmed that all 14 hub genes were differentially expressed in PDAC (Fig. 4). To verify the expression levels of the remaining 11 hub genes screened by GEPIA in a wider range of samples, a meta-analysis was performed based on the ONCOMINE database. As shown in Fig. 5, nine hub genes were significantly highly expressed in PDAC tissues including COL1A1, COL1A2, FN1, ITGA2, KRT19, LCN2, MMP9, MUC1 and VCAN. Finally, the protein expression levels of these nine hub genes were determined using the Human Protein Atlas (HPA) database, and all of them seemed to be highly expressed in PDAC tissues compared with normal pancreatic tissues (Fig. 6).

### Survival analysis

**Figure 7:**
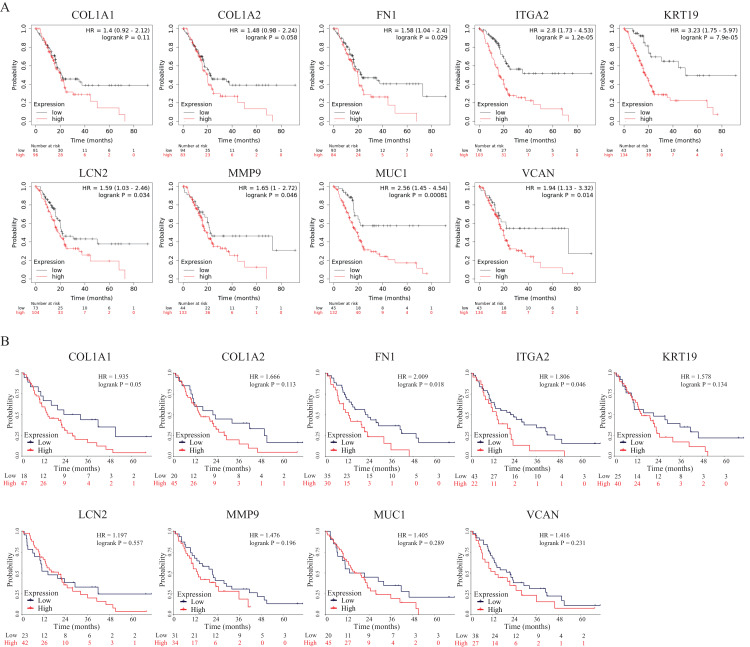
OS of the nine hub genes (COL1A1, COL1A2, FN1, ITGA2, KRT19, LCN2, MMP9, MUC1 and VCAN) in PDAC patients analyzed by (A) Kaplan–Meier plotter and the validation; (B) GEO dataset GSE62452. Log-rank *P* < 0.05 was considered as statistically significant. OS, overall survival; PDAC, pancreatic ductal adenocarcinoma.

To further explore the prognostic values of these nine hub genes, overall survival analyses were conducted by Kaplan–Meier plotter and the survival data of the validation dataset GSE62452. As shown in Fig. 7A, seven hub genes overexpressed in PDAC patients predicted poor prognosis including FN1 (Hazard ratio (HR) = 1.58, *p* = 0.029), ITGA2 (HR =2.8, *p* = 1.2E−05), KRT19 (HR = 3.23, *p* = 7.9E−05), LCN2 (HR = 1.59, *p* = 0.034), MMP9 (HR = 1.65, *p* = 0.046), MUC1 (HR = 2.56, *p* = 0.00081) and VCAN (HR = 1.94, *p* = 0.014), while there were no significant differences in COL1A1 (HR = 1.4, *p* = 0.11) and COL1A2 (HR = 1.48, *p* = 0.058). However, in the survival analysis results of the validation GEO dataset, only two genes (FN1 (HR = 2.009, *p* = 0.018) and ITGA2 (HR = 1.806, *p* = 0.046)) indicated significantly poor prognosis, while there were no significant differences in other seven genes (COL1A1 (HR = 1.935, *p* = 0.05), COL1A2 (HR = 1.666, *p* = 0.113), KRT19 (HR = 1.578, *p* = 0.134), LCN2 (HR = 1.197, *p* = 0.557), MMP9 (HR = 1.476, *p* = 0.196), MUC1 (HR = 1.405, *p* = 0.289) and VCAN (HR = 1.416, *p* = 0.231)) (Fig. 7B). To our knowledge, the mechanistic role of FN1 in pancreatic cancer has not been studied, so FN1 was chosen for further analysis in PDAC cells.

### Depletion of FN1 inhibited cell proliferation, blocked the cell cycle and induced apoptosis in PDAC cells

**Figure 8:**
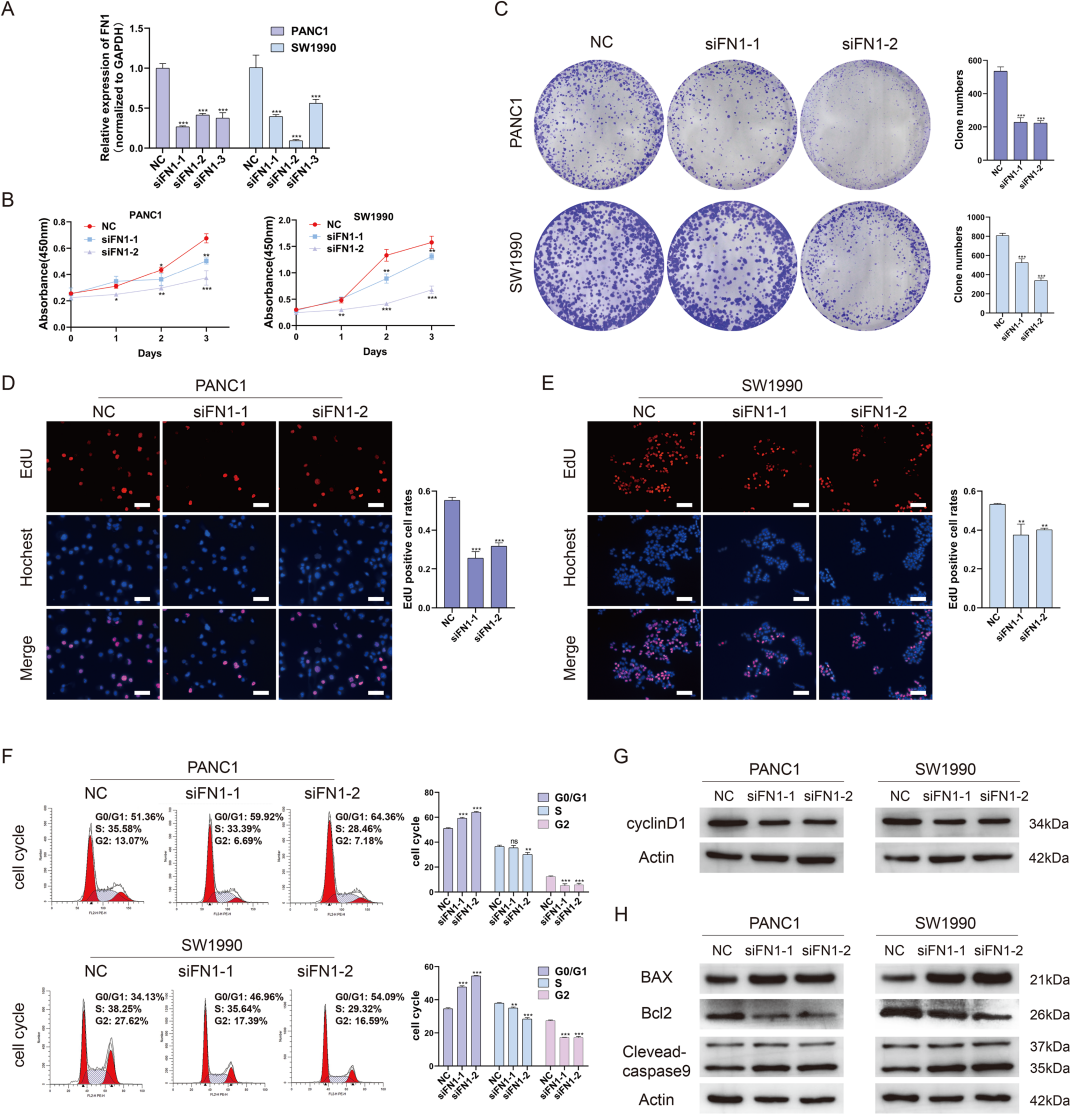
Depletion of FN1 inhibited cell proliferation, blocked cell cycle and induced apoptosis of PDAC cells. (A) The knockdown efficiency of the siRNAs against FN1 was confirmed by qRT-PCR. (B–E) CCK-8, colony formation and EdU assays were performed to evaluate the proliferation of the PDAC cell lines. (F, G) Cell cycle was analyzed by flow cytometry and western blot. (H) Cell apoptosis marker Bax, clevaead-caspase9 and Bcl2 were analyzed by western blot. PDAC, pancreatic carcinoma; CCK-8, Cell Counting Cit-8; EdU, 5-Ethynyl-2′-deoxyuridine. Scale bar = 50 μm. *P < 0.05, **P < 0.01, ***P < 0.001.

**Figure 9:**
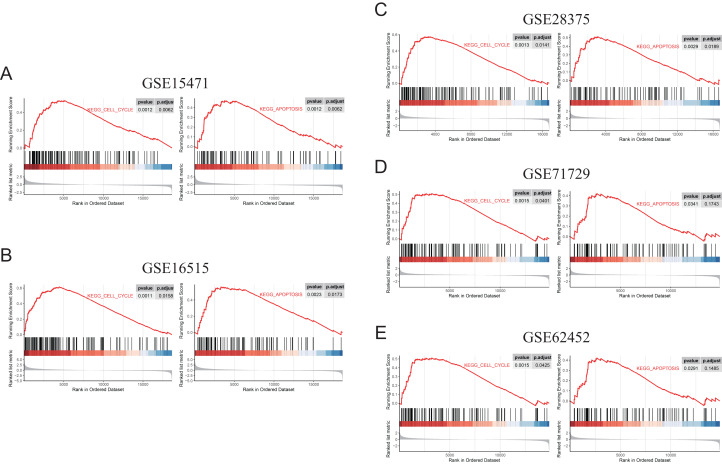
GSEA (gene sets “KEGG_CELL_CYCLE” and “KEGG_APOPTOSIS”) results of discovery GEO datasets and the validation GEO dataset. (A) GSEA results of GSE15471. (B) GSEA results of GSE16515. (C) GSEA results of GSE28375. (D) GSEA results of GSE71729. (E) GSEA results of GSE62452.

To further elucidate the role of FN1 in pancreatic carcinogenesis, we knocked down FN1 using small interfering RNA (siRNA) in PANC1 and SW1990 cell lines. The knockdown efficiency of FN1 was confirmed using qRT-PCR (Fig. 8A). The results showed that siFN1-1 and siFN1-2 had better knockdown effects and were selected for further studies. Colony formation assays and CCK-8 assays suggested that FN1 downregulation significantly inhibited the proliferation abilities of PANC1 and SW1990 cells (Figs. 8B, 8C). Consistent with these results, EdU assays showed that cell viability was inhibited in FN1-downregulated PDAC cells (Figs. 8D, 8E). We next explored the effects of FN1 on the cell cycle using flow cytometry and western blotting. The cell cycle was obviously arrested in G0/G1 phase, and the expression of CyclinD1, which promotes cell cycle progression, was significantly decreased after FN1 knockdown (Figs. 8F, 8G). Finally, apoptosis-related proteins were detected by western blot. As illustrated by the results, FN1 knockdown significantly increased the expression of the proapoptotic proteins BAX and clevaead-caspase9, and decreased the expression level of the antiapoptotic protein Bcl2 (Fig. 8H). These results indicate that FN1 downregulation significantly inhibited PANC1 and SW1990 cell growth, blocked the cell cycle and induced apoptosis *in vitro*, which was further confirmed by GSEA (Fig. 9).

## Discussion

PDAC is a malignant tumor with poor prognosis, due to the lack of specific early diagnostic markers and effective therapeutic targets. To further clarify the pathogenesis of pancreatic cancer and identify effective therapeutic targets, integrated computational analyses based on a large number of clinical samples from different sources were conducted. In this study, four GEO datasets (GSE15471, GSE16515, GSE28735 and GSE71729) were analyzed as discovery datasets to screen DEGs that may play important roles in pancreatic carcinogenesis. Next, a PPI network was constructed, and GO annotation and KEGG pathway enrichment analyses of the DEGs were performed *via* the STRING database and R software. Then, hub genes were screened through an integrated analysis of the correlation between these DEGs, and their expression levels in PDAC were subsequently identified *via* the GEPIA, ONCOMINE, HPA databases and validation GEO dataset GSE62452. Nine genes (COL1A1, COL1A2, FN1, ITGA2, KRT19, LCN2, MMP9, MUC1 and VCAN) were considered hub genes, and all of them were significantly highly expressed in PDAC tissues at both the mRNA and protein levels. Furthermore, the prognostic values of these nine hub genes were examined by Kaplan‑Meier plotter and the validation GEO dataset GSE62452. In the prognostic analysis of the two groups, only ITGA2 and FN1 had significant predictive values.

ITGA2 is the alpha subunit of a transmembrane receptor for collagens and related proteins. Overexpression of ITGA2 promotes the proliferation and invasion of tumor cells, while its knockdown decreases the degree of these processes (Ren et al., 2019). ITGA2 has been reported to play a critical role in modulating the pancreatic cancer immune response by transcriptionally increasing the expression of PD-L1 in cancer cells (Ren et al., 2019). At the same time, it has been reported that ITGA2 is associated with unfavorable survival of PDAC patients by affecting the malignant biological behavior of pancreatic cancer cells (Rozengurt, Sinnett-Smith & Eibl, 2018; Shimomura et al., 2020; Yan et al., 2020). Considering that the mechanism of ITGA2 in PDAC has been studied partly, we focused on FN1 which has no mechanism study in PDAC for further study.

FN1 is a glycoprotein present in a soluble dimeric form in plasma and in a dimeric or multimeric form at the cell surface and in extracellular matrix. Fibronectin is involved in cell adhesion and migration processes, including embryogenesis, wound healing, blood coagulation, host defense, and metastasis (Glasner et al., 2018; Griffith et al., 2006). Recent studies have shown that FN1 plays an important role in the occurrence and development of a variety of tumors (Ding, Liu & Lai, 2020; Lee et al., 2015; Yoshihara et al., 2020; Zhan et al., 2018; Zhou et al., 2019). For example, FN1 was highly expressed in sporadic medullary thyroid cancer tissues, and the immunohistochemical score of FN1 in tumor tissues was negatively correlated with American Joint Committee on Cancer stage, tumor histopathological typing and lymph node classification (Zhan et al., 2018). Peritoneal dissemination of ovarian cancer induces intercellular interactions through the FN1/AKT signaling pathway and induces the reduction in platinum sensitivity in ovarian cancer cells (Yoshihara et al., 2020). The FN1 gene may provide an oligomerization domain of its constituent active promoter and encoding protein to overexpress and promote the activation of the FGFR1 kinase domain in mesenchymal tumors (Lee et al., 2015). Furthermore, there are also some studies reported that FN1 is highly expressed in PDAC, but its mechanistic role of FN1 in PDAC remains fully unknown (Ding, Liu & Lai, 2020; Zhou et al., 2019).

Based on this knowledge, we focused on FN1, which has both significant differential expression and remarkably significant prognostic value in PDAC. In our study, the biological functions of FN1 in PDAC growth were validated in the pancreatic cancer cell lines PANC1 and SW1990. Knockdown of FN1 in PDAC cell lines through siRNA inhibited cell proliferation and colony formation rates. The results provide strong support for our computational analyses, which suggests that FN1 may be a potential biomarker for the selection of therapeutic strategies for PDAC. Regarding the mechanism, knockdown of FN1 promotes cell apoptosis and induces cell cycle arrest by reducing the expression of cyclin D1, indicating that FN1 contributes to the development of pancreatic cancer by affecting cell apoptosis and the cell cycle in a certain manner.

## Conclusions

In summary, two remarkable hub genes (FN1 and ITGA2), that are abnormally overexpressed and predict a poor prognosis in PDAC patients were identified in our study based on a large number of clinical samples. In addition, for the first time, we highlighted the association between FN1 and cell viability, apoptosis and the cell cycle in pancreatic cancer cells. FN1 might be a novel biomarker for therapy and prognostic prediction in patients with PDAC. More importance should be attached to FN1 and other hub genes in further studies of PDAC, and further explorations into relative molecular mechanisms should be performed.

## Supplemental Information

10.7717/peerj.12141/supp-1 Supplemental Information 1 Uncropped Blots of Figure 8.

10.7717/peerj.12141/supp-2 Supplemental Information 2 Raw data for Figure 8.

## Additional Information and Declarations

### DNA Deposition

The following information was supplied regarding the deposition of DNA sequences: The data are available at NCBI: GSE15471, GSE16515, GSE28735, GSE71729 and GSE62452.

### Data Availability

The following information was supplied regarding data availability: Data are available in the Supplemental Files.
