# Supplementary figures and images for: Comprehensive analysis of abnormal expression, prognostic value and oncogenic role of the hub gene FN1 in pancreatic ductal adenocarcinoma via bioinformatic analysis and in vitro experiments
Source: PeerJ. 2021 Sep 6;9:e12141. doi: 10.7717/peerj.12141 (PMC8428264; doi:10.7717/peerj.12141)

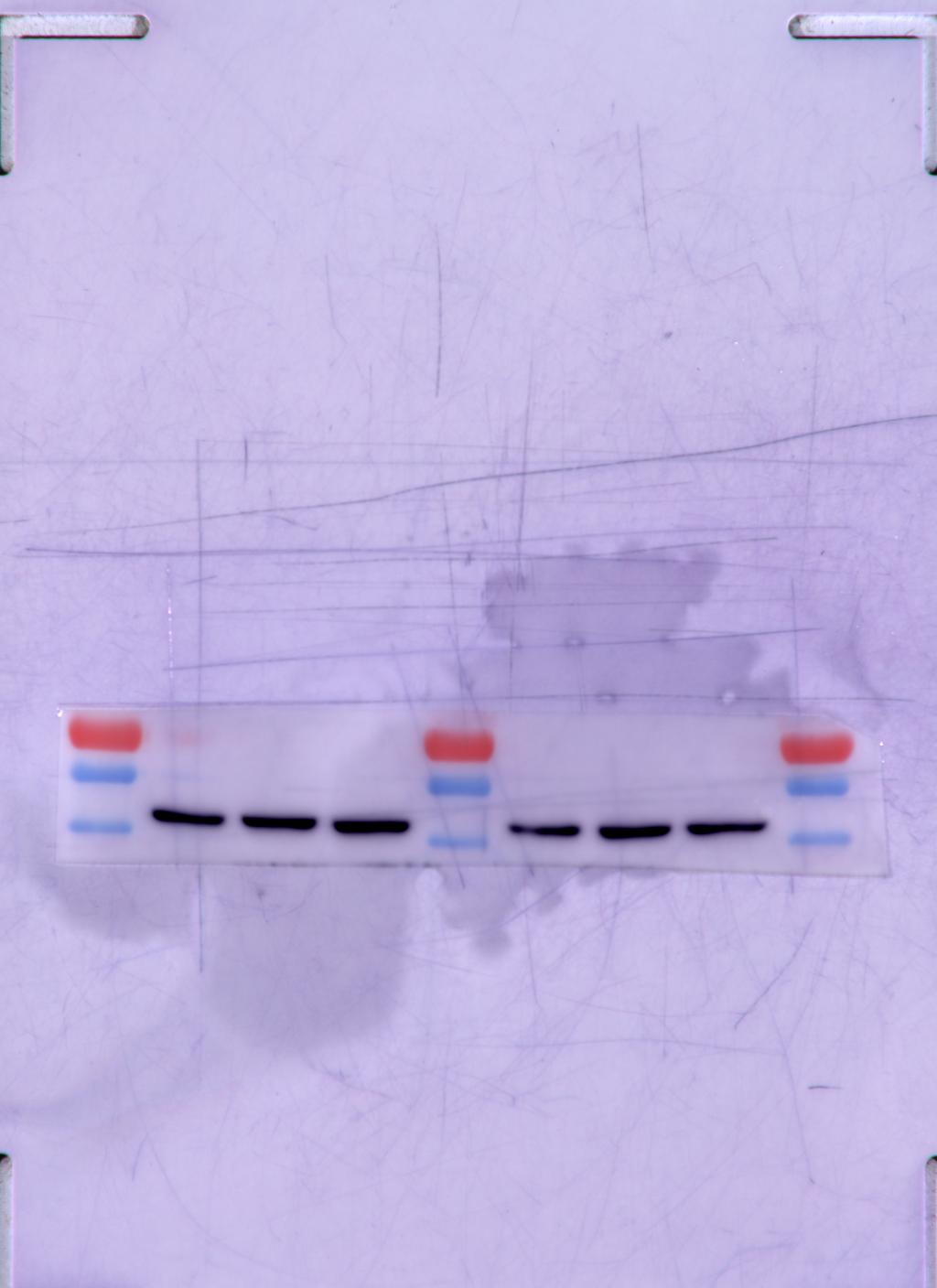

Supplement: Supplemental Information 1 [file peerj-09-12141-s001.zip › Uncropped Blots/figure 8G - actin.jpg]

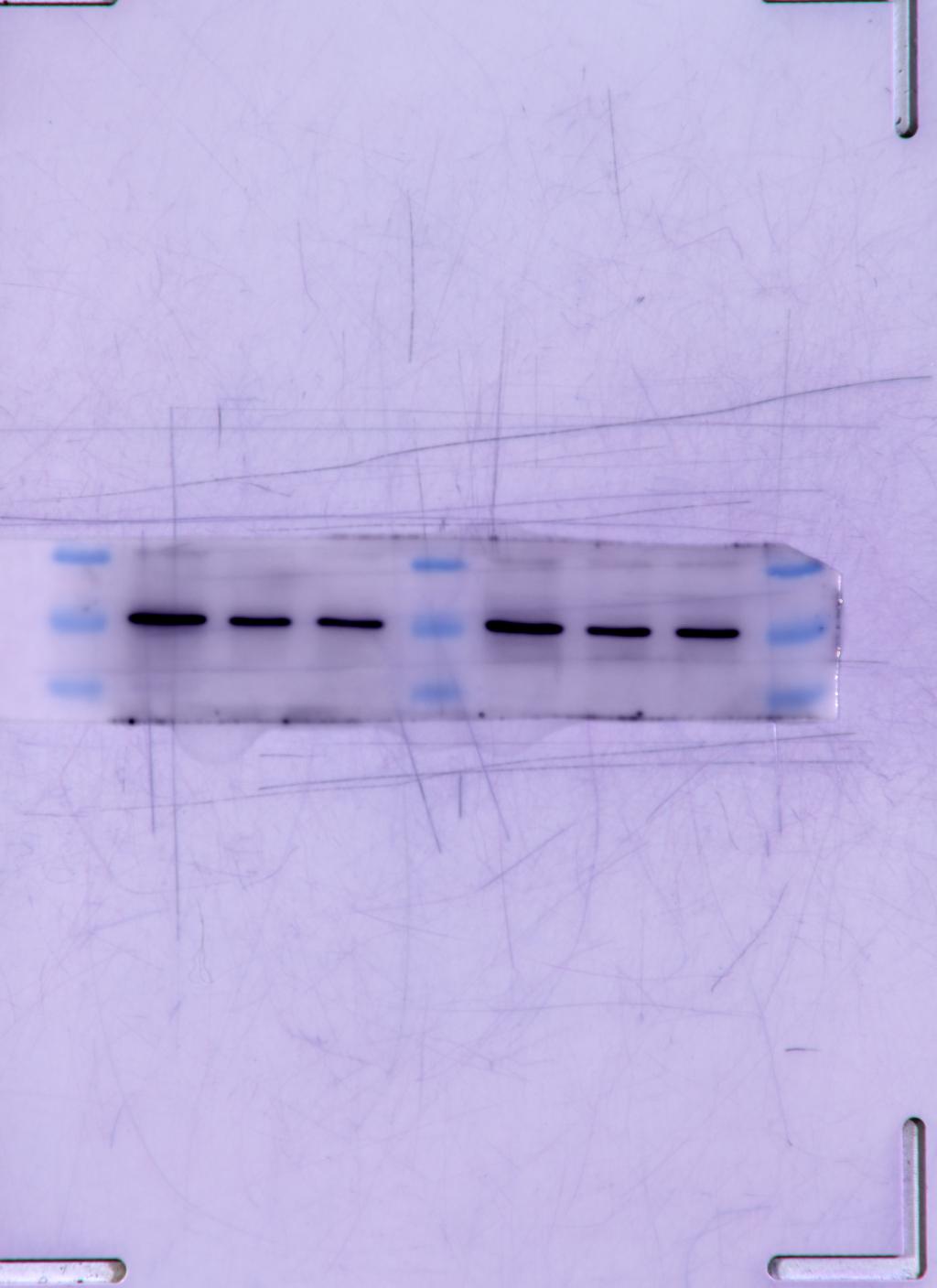

Supplement: Supplemental Information 1 [file peerj-09-12141-s001.zip › Uncropped Blots/figure 8G - cyclin D1.jpg]

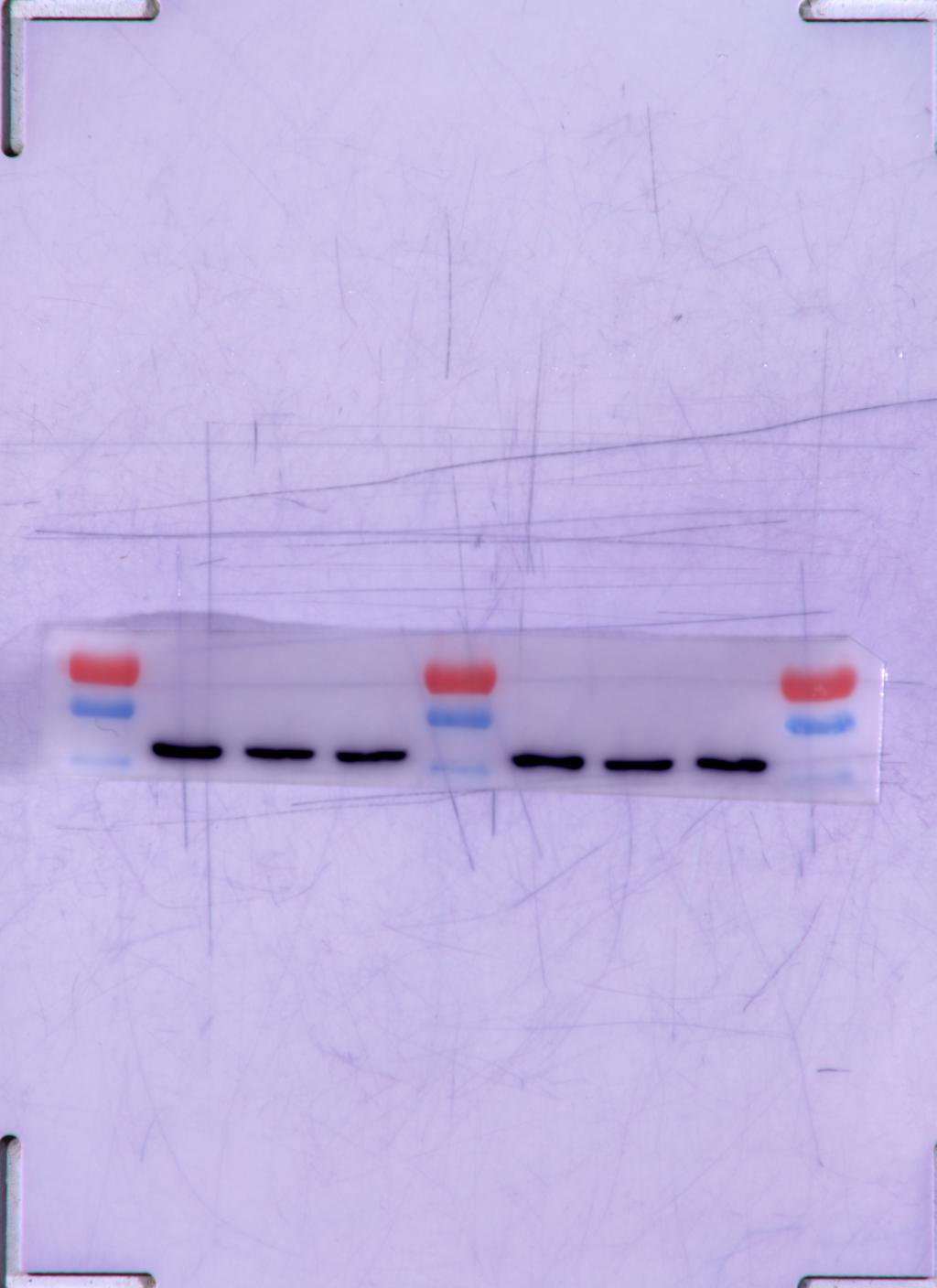

Supplement: Supplemental Information 1 [file peerj-09-12141-s001.zip › Uncropped Blots/figure 8H - actin.jpg]

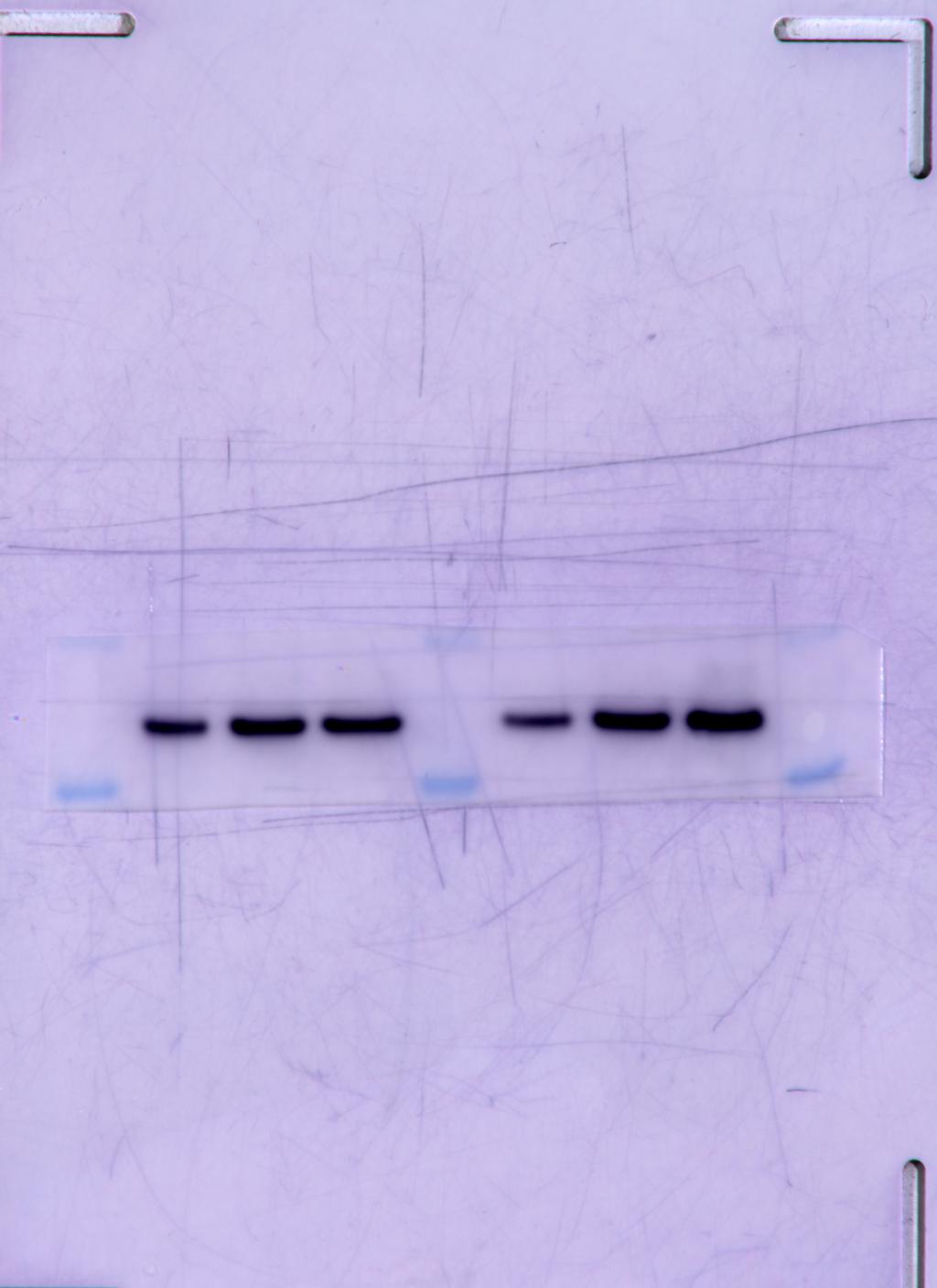

Supplement: Supplemental Information 1 [file peerj-09-12141-s001.zip › Uncropped Blots/figure 8H - BAX.jpg]

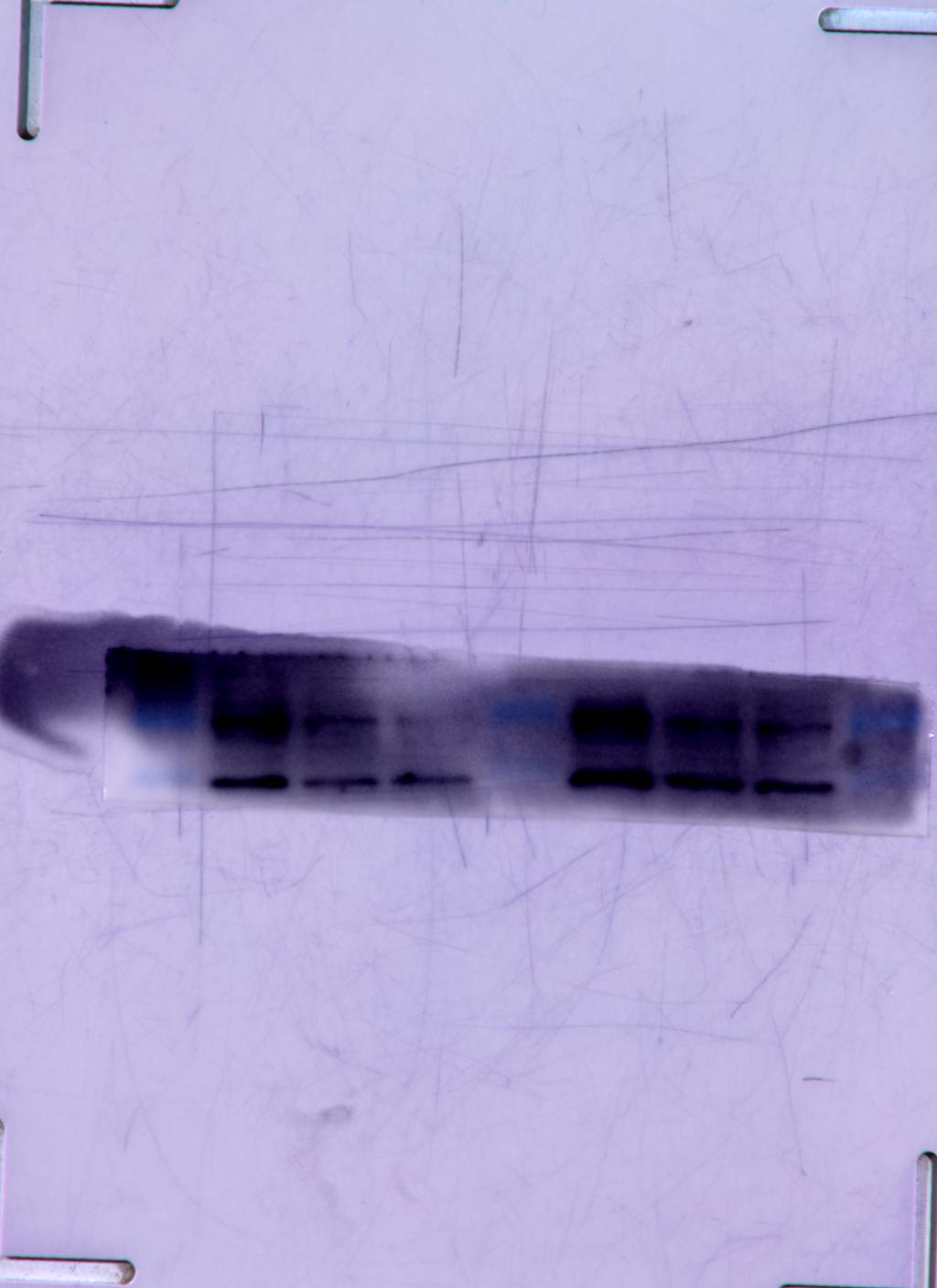

Supplement: Supplemental Information 1 [file peerj-09-12141-s001.zip › Uncropped Blots/figure 8H - Bcl2.jpg]

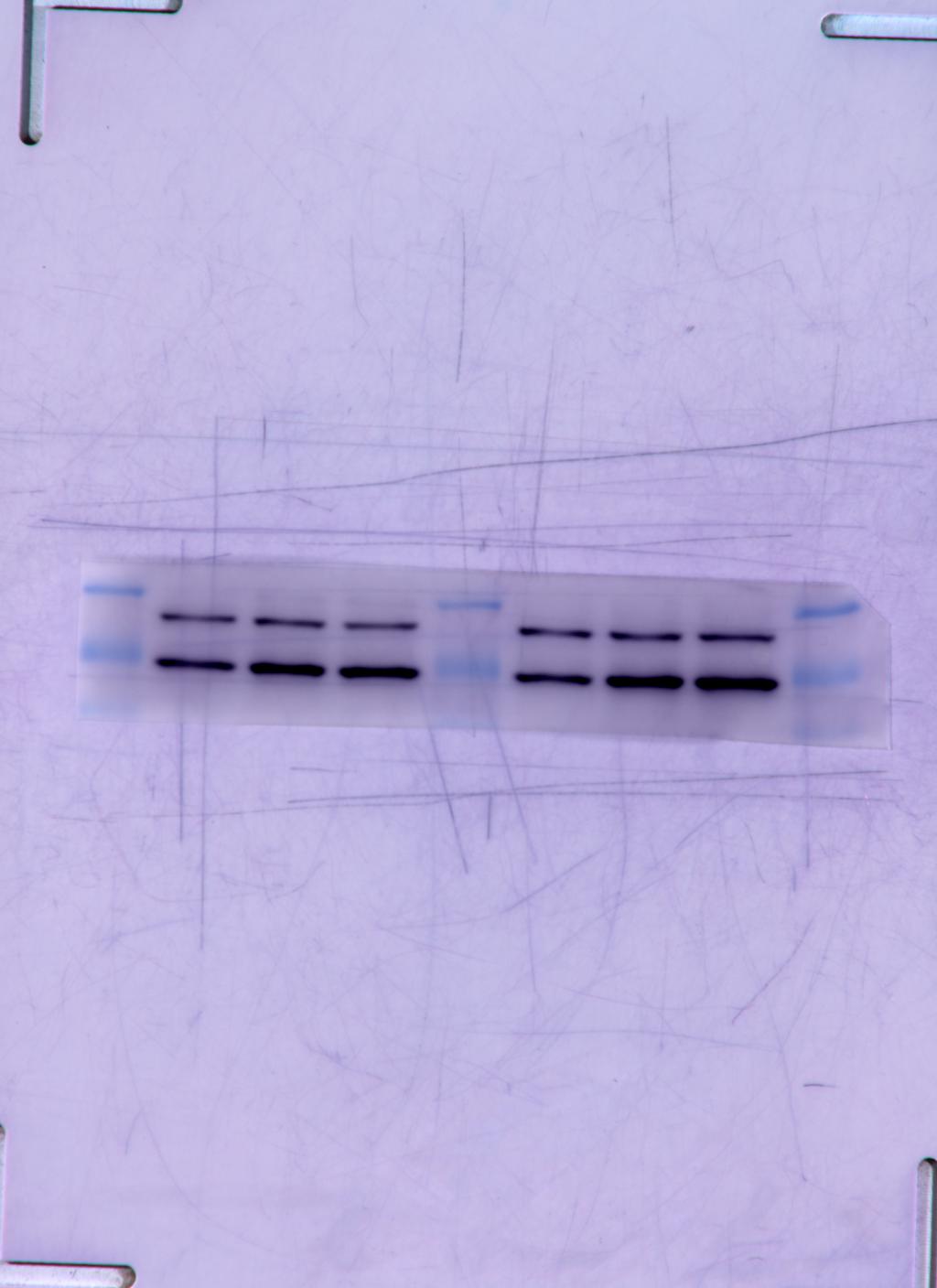

Supplement: Supplemental Information 1 [file peerj-09-12141-s001.zip › Uncropped Blots/figure 8H - clevead caspase9.jpg]
